# DCAF17 Mutation in Woodhouse–Sakati Syndrome: A Case Report on a Novel Homozygous Variant

**DOI:** 10.1155/crpe/9913412

**Published:** 2025-09-29

**Authors:** Asal Khalili Dehkordi, Rahim Vakili

**Affiliations:** ^1^Department of Pediatrics, School of Medicine, Growth and Development Research Center, Children's Medical Center, Tehran University of Medical Sciences, Tehran, Iran; ^2^Department of Pediatrics, Faculty of Medicine, Mashhad University of Medical Sciences, Mashhad, Iran

**Keywords:** autosomal recessive genetic disorder, DCAF17, hypogonadism, OMIM: 80067, Woodhouse–Sakati syndrome

## Abstract

**Background:** Woodhouse–Sakati syndrome (WSS) is a rare autosomal recessive disorder characterized by a constellation of symptoms, including alopecia, hypogonadism, diabetes, mental retardation, and extrapyramidal syndrome. Here, we present a case study of a girl with WSS, focusing on clinical features, genetic analysis, and treatment.

**Case Description:** The patient is a 16-year-old female who presented with primary amenorrhea and underdeveloped secondary sexual characteristics. She has first-degree consanguineous parents. Clinical evaluations, laboratory tests, whole-exome sequencing, and karyotyping were performed to diagnose WSS. The patient exhibited notable frontotemporal alopecia, hypogonadism, and intellectual decline. Genetic analysis revealed a homozygous mutation (c.1001 + 1G > A) in the DCAF17 gene, a known causative gene of WSS. In addition to hormone therapy to induce puberty, the patient was referred to neurology for further evaluation.

**Conclusions:** This case highlights the importance of considering WSS in patients with alopecia, hypogonadism, and consanguineous backgrounds. Genetic testing plays a crucial role in diagnosis, while hormone therapy may alleviate some symptoms. WSS is a complex syndrome with varied clinical manifestations, necessitating multidisciplinary treatment. Early recognition and effective management are essential for improving the quality of life of affected individuals.

**Summary**

• WSS is a hereditary disorder causing alopecia and hypogonadism.
• The presented case is a teenage girl diagnosed by clinical evaluations and WES.

## 1. Introduction

Woodhouse–Sakati syndrome (WSS) (MIM 241080) is an uncommon autosomal recessive disorder initially observed within a few consanguineous Saudi families in 1983. This syndrome is primarily represented by alopecia, hypogonadism, diabetes mellitus, mental retardation, and extrapyramidal syndrome [1].

Additional endocrine manifestations encompass reduced levels of insulin-like growth factor 1 (IGF-1) among all patients, with up to 40% of the individuals suffering from hypothyroidism [2].

The neurological manifestations of the disease are progressive and mainly involve extrapyramidal movements (such as dystonic spasms accompanied by dystonic posturing, dysarthria, and dysphagia), an intellectual decline that ranges from mild to severe, and bilateral postlingual sensorineural hearing loss [2, 3].

Alopecia frequently manifests during the early stages of childhood in affected individuals, potentially affecting various areas, such as scalp hair, eyebrows, eyelashes, and pubic and axillary hair [4].

The etiologic basis of this condition can be linked to the occurrence of homozygous pathogenic mutations in the DCAF17 gene (DDB1 and CUL4-associated factor 17), also commonly known as C2orf37. This gene is located on Chromosome 2q22.3-q35. The mutation of DCAF17 hinders the normal functioning of the nucleolar protein, which has the potential to serve as a substrate receptor for the CUL4-DDB1 E3 ubiquitin–protein ligase complex, leading to errors in regular cell processes, such as cell cycle regulation, cell senescence, and apoptosis. These disruptions could be the underlying cause of the pathophysiology of WSS [5].

In addition, the establishment of a founder effect was also observed. Calculations using a simplified likelihood algorithm indicate that this mutation occurred around 55 generations ago [5, 6]. It is worth noting that the existing literature on WSS primarily focuses on its occurrence within consanguineous families. Besides, consanguinity has been suggested as a potential risk factor for WSS [7].

We share the second report on WSS from Iran in this context [8]. The patient under study is a 16-year-old girl presenting with amenorrhea and undeveloped secondary sexual characteristics.

## 2. Case Presentation

The present case is a 16-year-old female patient referred to our clinic by a gynecologist regarding her underdeveloped secondary sexual characteristics and primary amenorrhea.

She is the only child of a first-degree consanguineous couple. She had a full-term pregnancy and delivered vaginally without complications. During her childhood, she experienced normal developmental milestones with no notable deviations. However, she had difficulties in speech and language development, and her school performance was below average compared to that of her typically developing peers. She faced some challenges in her school years, which may have been influenced by her speech and language difficulties.

**Figure 1:**
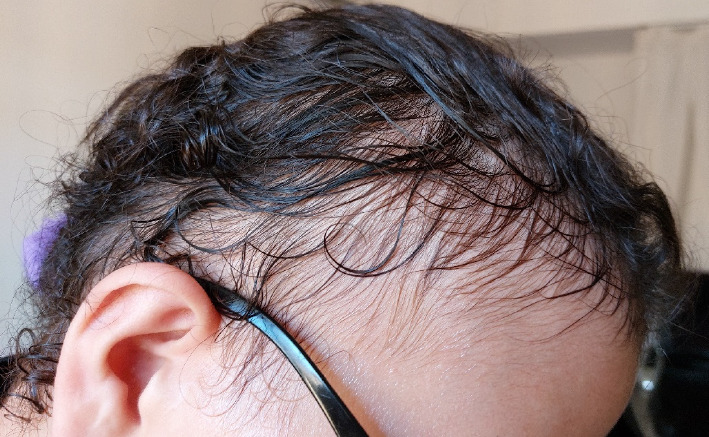
Clinical photograph of frontotemporal alopecia.

On examination, she is 172 cm tall (above the 92nd percentile) and weighs 86 kg (above the 97th percentile). She has significant frontotemporal alopecia, which first manifested during childhood and has since progressed throughout the years (Figure 1). She had underdeveloped secondary sexual characteristics, with Tanner Stage 1 breasts and pubic hairs. The external genitalia are feminine. The results of the visual, auditory, and dental evaluations, as well as the remainder of the examination, were within norms. There are no apparent motor movement abnormalities.

She had no history of swallowing difficulty, seizures, abnormal limb posturing or movement, or postural disfiguring features.

Upon investigation, elevated levels of follicle-stimulating hormone (FSH) and luteinizing hormone (LH), alongside decreased estradiol levels (0.1 pg/mL), were observed, indicating the presence of hypergonadotropic hypogonadism. The serum prolactin level was within normal limits. Thyroid hormone tests showed a normally functioning thyroid.

Pelvic ultrasound showed hypoplastic ovaries and uterus. The uterus's dimensions were 28 ∗ 7 mm, indicating a small size with a thin thickness. Likewise, the ovaries were small, and no apparent follicles were detected.

### 2.1. Diagnosis

The patient's presentation of primary amenorrhea, gonadal dysgenesis, alopecia, and mild intellectual decline, in conjunction with the consanguineous marriage of her parents, led to suspicion of a potential genetic cause. Thus, the patient underwent whole-exome sequencing and a karyotype test. The sample for the karyotype was heparinized blood, and the analysis was conducted using 15 metaphase spreads with the GTG technique, achieving a band resolution of 400–440 bands.

She had a normal 46, XX karyotype. Genetic analysis confirmed the presence of the likely pathogenic mutation NM_025000.4 c.1001 + 1G > A in the homozygous DCAF17 gene.

**Figure 2:**
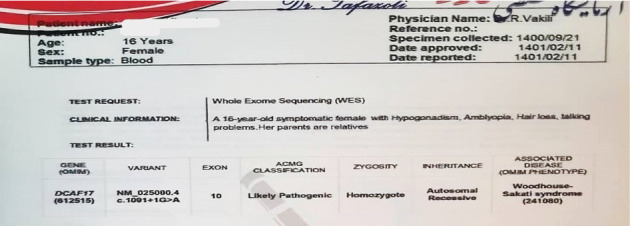
WES results confirming the mutation.

The variant NM_025000.4 c.1001 + 1G > A in the DCAF17 gene has not been documented in the current scientific literature. The mutation is likely to disrupt RNA maturation, specifically affecting the processing of Exon 10 (Figure 2).

### 2.2. Treatment

Conjugated estrogen and medroxyprogesterone were administered to induce puberty.

The patient was referred to a neurology specialist for a thorough neurological assessment. Periodic evaluations of thyroid function and blood glucose levels were also recommended.

## 3. Discussion

We went through an evaluation of a case of WSS, characterized by symptoms of alopecia and hypogonadism. We assessed the various manifestations, laboratory findings, and results of genetic tests.

WSS is an autosomal recessive disorder that may represent a novel neuroendocrine ectodermal syndrome, given its diverse manifestations [2]. The key genetic finding in WSS is the discovery of a biallelic founder variant in the C2orf37 gene, specifically DCAF17, which encodes a nucleolar protein. The gene exhibits expression in the brain, skin, and liver, which is consistent with the involvement of these organs in individuals with WSS [4]. Most mutations in the DCAF17 gene result in a nucleolar protein with atypical characteristics, such as shortened length, instability, rapid degradation, or impaired physiological function. The clinical manifestations of WSS appear to result from the absence of this protein's activity [9].

Various combinations of abnormalities have been reported in patients with WSS, including neuroectodermal manifestations, sensorineural deafness, diabetes, endocrine abnormalities, extrapyramidal signs, keratoconus, syndactyly of the hands and feet, progeria, thymus and cardiac anomalies, anodontia, polyneuropathy, and seizures [1–5, 10].

Alopecia is a prominent feature of WSS and typically manifests as partial and progressive hair loss, particularly in the temporal areas. It is also marked by sparse eyebrows and eyelashes [2].

Hypogonadism is a consistent feature, observed in nearly all patients, with both hypogonadotropic and hypergonadotropic forms documented. In some cases, the distinction could not be clearly defined or interpreted. Our patient was diagnosed with hypergonadotropic hypogonadism. This condition often manifests when there is a delay or absence of pubertal development. Patients exhibit early Tanner staging and absence of secondary sexual characteristics among females, which typically prompts consultation during adolescence [1, 11].

Low levels of IGF-1 are a consistent biochemical abnormality observed in Al-Semari et al.'s study across all cases. This finding suggests that a potential primary hormonal error in this syndrome may be a low IGF-1 level [2].

Hypothyroidism affects 30% of patients with WSS, usually around age 20 [11]. In addition, diabetes mellitus is common among individuals with WSS, with a prevalence of up to 66%. It primarily manifests in the age range from adolescence to early adulthood [3, 5].

Neurological manifestations, including facio-cervical dystonia affecting the oropharyngeal muscles, have been comprehensively described in Schneider et al.'s study. The initiation of neurological symptoms typically occurs at approximately 20 years of age, which might explain their absence in our case. The typical course of the disorder demonstrates a sequential development of symptoms leading to generalized dystonia and choreoathetosis movements, which eventually result in issues with posture and gait. The previously mentioned symptoms are observed in 42% of cases and 89% of patients aged 25 years and older [12].

Currently, the therapeutic approach for WSS primarily focuses on managing symptoms and providing palliative care [2]. The administration of exogenous hormone therapy, as demonstrated in our discussed case, has the potential to facilitate the development of secondary sex characteristics in affected individuals [9].

This study presents a novel mutation that offers new insights into our understanding of WSS and expands the genetic spectrum associated with this disorder. However, one limitation of this report is the lack of confirmation of the parental carrier status, which could have significantly strengthened the evidence for the pathogenicity of the novel variant. In addition, the posttreatment status of the patient has not been reported, limiting our ability to assess the long-term effects and outcomes of the intervention.

## 4. Conclusion

The presented case of WSS highlights the complexity of this rare autosomal recessive disorder. The combination of diverse clinical features, such as alopecia, hypogonadism, diabetes mellitus, and extrapyramidal signs, forms a complex picture that mandates a multidisciplinary approach to diagnosis and treatment. The DCAF17 gene, which encodes an atypical nucleolar protein, plays a role in the pathogenesis of WSS. Notably, consistent biochemical abnormalities, such as low IGF-1 levels, provide additional insight into the hormonal dynamics of this syndrome. The treatment of WSS focuses on symptom relief and enhancing the quality of life for those affected. This case illustrates the significance of comprehensive assessment, genetic analysis, and targeted interventions for managing WSS's various symptoms.

## Data Availability

The data that support the findings of this study are available from the corresponding author upon reasonable request.
